# Employees’ experiences of a large-scale implementation in a public care setting: a novel mixed-method approach to content analysis

**DOI:** 10.1186/s12913-024-10560-9

**Published:** 2024-01-18

**Authors:** My Säfström, Ulrika Löfkvist

**Affiliations:** 1 Primary care and health, Uppsala County Council, Uppsala, Sweden; 2 Department of Public Health and Caring Sciences, Uppsala University, Uppsala, Sweden

**Keywords:** Implementation, Organisational change, Employee experience, Public care, Organisational and social work environment, Disability

## Abstract

**Background:**

Research for evidence-based interventions and strategies for implementation continues. Yet there is a continued shortage of qualified health care staff while stress and burnout are common. Health care professionals’ individual perceptions towards change needs to be considered to succeed in organisational change. It is therefore relevant to investigate how implementation processes affect employees within the health care sector. Challenges to implementation are especially large in the field of disability care. The present study aims to investigate employees’ experiences of an ongoing large-scale implementation, and what they perceived as important to succeed in a complex clinical setting.

**Methods:**

Semi-structured focus group interviews were conducted with a self-selected sample of employees from a large and complex health care organisation responsible for public disability care in a centrally located Swedish region. A mixed-method approach adapted to content analysis was performed in a three-step process. In the first round, each unit of analysis was selected and then colour coded. In a second round, the coloured units were coded according to content analysis, and categories and concepts were compared and adjusted until the two researchers reached consensus. Finally, to further complement the content analysis, a quantitative analysis of the colour categories was made.

**Results:**

In general, employees experienced the implementation as being insufficient, yet opinions of the process of implementation were mixed. Most positive experiences were found in relation to the outcomes that the new method had on work effectiveness and patient care. Closely related topics like time constraints, uncertainties concerning the method and the need for supportive functions reoccurred in several concepts suggesting a relationship between differing contextual factors, implementation activities and fidelity. Also evident in the results were the strain on organisational and social work environment and the importance of managers’ active leadership.

**Conclusions:**

Implementation processes are experienced as challenging for employees. Key facilitators are available support functions, clear leadership and time that is sufficient and kept sacrosanct. Leaders need to communicate how and why employees may experience implementation processes differently. The impact that organisational change has on work environment should be considered.

**Supplementary Information:**

The online version contains supplementary material available at 10.1186/s12913-024-10560-9.

## Background

The road from research to efficient use of new methods in routine health care is usually long and hard [1]. Meanwhile, the health care system is undergoing many changes to cope with an aging population with more complex care needs, making the need for more cost-effective treatments and to streamline working methods greater than ever [2–5].

Still it is a common occurrence that implementations end in failure [6]. Several theories have been put forward over the years to provide better understanding and guidance in the implementation process [7]. However, implementation in clinical practice is complex. When implementing new methods there has been a long-standing debate between the need to on the one hand adhere to treatment integrity, and on the other to make changes in order for the intervention to fit a specific context or with particular patients [8]. Von Thiele Schwartz, Aarons & Hasson (2019) theorizes that implementation success can be defined as the ability to optimize value across different levels and stakeholders [9]. Thus, aggregating the differing needs of patient, health care provider, organisation, and system. Von Thiele Schwartz and colleagues (2019) names this The Value Equation where implementation strategies increase fit either by optimizing the context so that it fits the intervention or by deliberate changes to the intervention, while still adhering to its core components, so that it fits the context [9].

Meanwhile as research for new evidence-based interventions and strategies for implementation continues there is a continued shortage of qualified health care staff [10, 11], and international comparisons show high levels of stress and burnout among primary care physicians [12]. It is therefore relevant to wonder how implementation processes affect employees within the health care sector. A systematic literary review found that health care professionals’ individual perceptions towards change needs to be considered in order to succeed in organisational change. Yet this research trajectory appeared to be undeveloped in healthcare [4].

Granberg et al. (2021) investigated managers’ experiences of implementation and note that the challenges to implementation are especially large in the field of disability care [13]. The authors mention the extensive and varied needs among patients as one possible reason. Ensuring comprehensive disability care is complex, requiring a team of professionals from different medical fields, creating a unique and special workplace culture. One such example is a public health care organisation located in a large central Swedish region. The organisation is responsible for the care of patients of all ages with different types of neurodevelopmental and mobility disorders. The care is organised in seven different clinical departments. Multi-disciplinary teams work with habilitative interventions, focusing mainly on psychosocial, educational, and therapeutic approaches. One of the departments has a medical focus but works exclusively in a supporting manner towards the other departments with no patients solely their own. The departments differ from each other in several aspects. The teams vary in size. Either a first or middle manager can conduct leadership. Finally, departments can specialize in a specific disability and be situated in the main city of the region or be responsible for several disabilities at once and be situated in one of the regions outer cities. The result being a complex organisation with differing needs on many levels and with multiple stakeholders.

This organisation is no exception to the changing landscape of health care and has experienced several changes in its organisation and work processes in recent years. One such is the implementation of a new method of documenting and revising patients’ care plans with the ambition of making each one a living document. The previous method relied on yearly renewal, while the new method focuses on continuous updates as patients’ care and context changes. The implementation affects the entirety of the organisation, with daily effects to the routines of about 200 employees, depending on their unique placements in the organisational context. For details on the implementation see Supplementary Material 1.

As there are few studies on individuals’ perceptions of organisational change in health care [4] the aim of the present study was to investigate employees’ experiences of this ongoing large-scale implementation, and what they perceive as important to succeed in a complex clinical setting. Special interest was taken in finding patterns of perceived strengths or challenges that affected the implementation process. Research question: How do the employees of a complex health care organisation experience the implementation of a new working method?

## Methods

The current study used a mixed-method approach to content analysis to investigate participants experiences of an ongoing implementation process [14, 15]. During the preparation phase previous knowledge from literature and related practice helped formulate the semi-structured open-ended questions. In the organization phase a combination of deductive and inductive content analysis was used. An inductive approach was used during the data analysis and reporting phase. Finally, the main descriptive features of the formulated concepts were quantitatively calculated. Semi-structured focus group interviews were conducted with a self-selected sample of employees from the seven before mentioned departments [16]. Findings are reported in line with Standards for Reporting Qualitative Research (SRQR; see Supplementary Material 2) [17].

### Procedure

All clinical employees without leadership assignments were eligible for participation. Information about the study was given via email and during weekly department meetings. An estimated 140 people were reached this way. Participants who first volunteered were enrolled in the study after signing an informed consent. Managers had no say in who was accepted into the study, nor were they informed of when the interviews took place. The aim was to recruit one participant from each department. Recruitment ended when this goal was reached (*n* = 7). However, to enable participation by all, interviews needed to be held at two occasions, the first larger group in March of 2022 (*n* = 5; 80 min long) and the second smaller group one month later (*n* = 2; 30 min long). Nearly all clinical professions were represented (social worker, psychologist, occupational therapist, physiotherapist, special education teacher and nurse), the only exceptions being speech language pathologist and dietician. Average time of employment was 4 years (SD = 1, 8).

To promote easier participation the interviews were conducted via video meetings as this has been proven to be a valid alternative in qualitative research [18]. To avoid online recording through Microsoft Teams, an external video camera was aimed at the computer screen to record the interviews. The lead researcher (MS) was at the time of data collection an employed clinician in one of the departments and had provided implementation support to management during the first few months of the implementation process. The interviews were therefore performed by the secondary researcher (UL) with whom the participants were unfamiliar, and with MS as observer (muted and with video off). The interview guide consisted of open-ended questions related to the research question. Focus was on participants’ experience of the implementation process, including the new method, their working conditions and the support received (see Supplementary Material 3).

### Data analysis

The recordings were transferred from the video camera and kept on an encrypted USB-drive. The interviews were then transcribed verbatim at which time individual statements were coded. The code key was also encrypted and stored separately from the recordings which were finally deleted after transcription was complete. The qualitative content analysis approach was employed by each researcher independently to discern central concepts. Two rounds of consensus were applied. In the first round, each unit of analysis was selected and then colour coded into one of five categories according to its general content related to the research questions: Green for positive statements. Red for negative statements. Yellow for neutral statements but in reference to the implementation. Blue for statements related to other insights that occurred because of the implementation process. Finally white for unspecified statements (not related to the implementation). The chosen units of analysis and colouring of each researcher were then compared and discussed to reach consensus in units and colouring. In a second round, the coloured units of analysis were coded in accordance with Elo & Kyngäs (2008) [14]. The categories and concepts were compared and adjusted until consensus was reached. To further complement the content analysis, a quantitative analysis of the colour categories was made [15]. By summing-up the total number of units of analysis within each concept, the size of each concept was determined. The distributions of the different colour categories were then calculated within each of the respective concepts.

This approach was created by the authors as an attempt to get a better overall understanding of strengths and barriers within implementation processes. By colouring all units of analysis before the traditional coding [14] both positive and negative utterances were flagged. Statements that were not directly related to the implementation process were identified early thus figuratively giving the opportunity to pan for gold in the material. When the units had been coloured and coded the material was quantified to give a better overview and understanding of the different concepts and which parts of the implementation process that employees experienced as strengths or challenges.

## Results

**Fig. 1:**
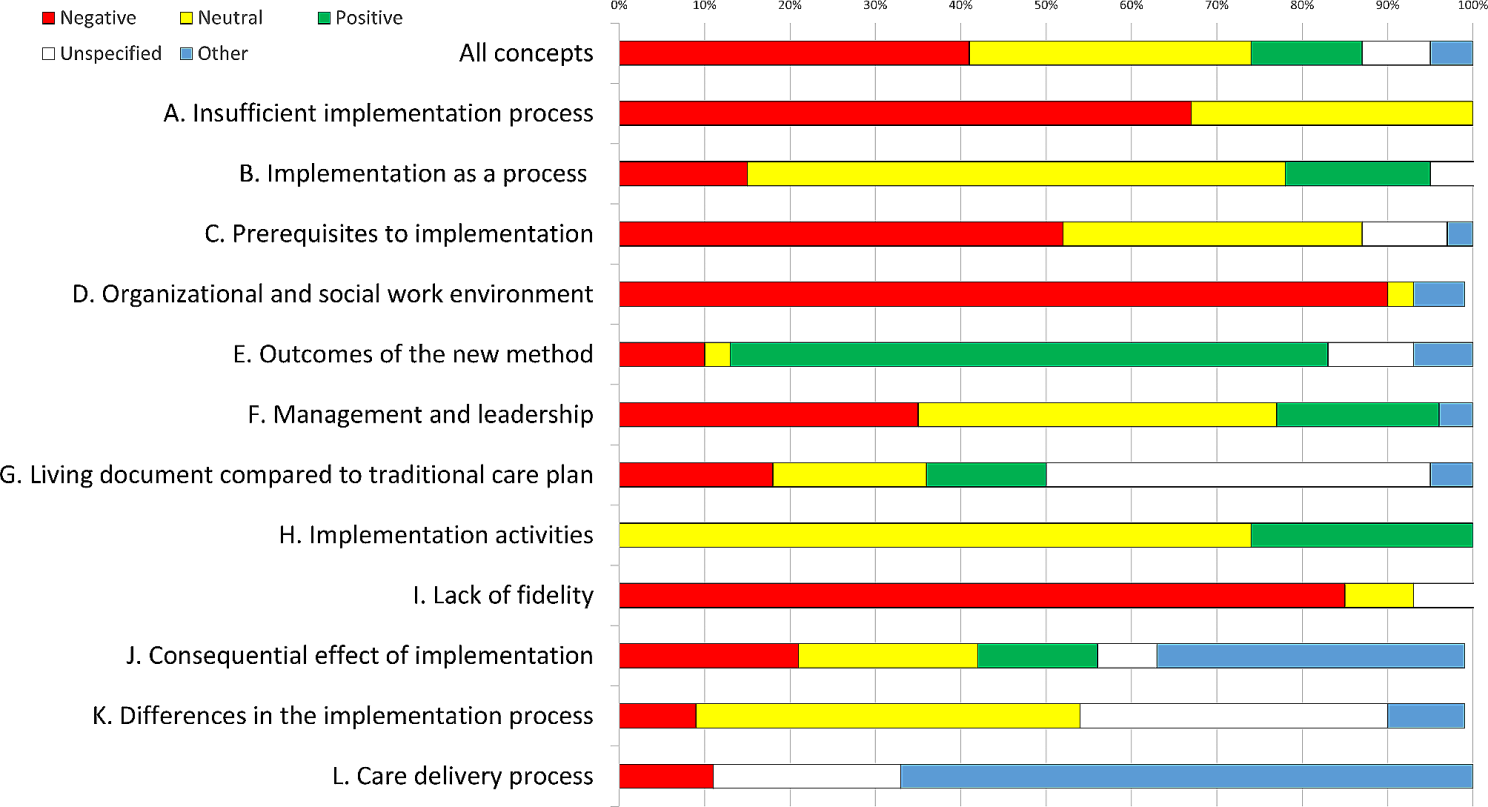
Concepts according to size (larger to smaller) with distribution of colour categories

The aim of the present study was to investigate employees’ experience of the implementation process. The result comprised of 348 units of analysis. Twelve concepts emerged (A–L). All are presented according to size together with their respective distribution of colour categories in Fig. 1. For precise numbers see Supplementary Material 4. Exemplifying quotes are presented in Table 1.

**Table 1:** Exemplifying quotes for main concepts. Respondent and corresponding colour category in parentheses

**A. Insufficient implementation process**
“And also we have co-workers who came about a year ago who kind of haven’t even realized yet that we’re doing this.” (No. 3, red.)
“It becomes more so that everyone sits around and tries, you try to help, I try to help someone even though I don’t know myself. Eh it gets more like that”. (No. 4, yellow.)
**B. Implementation as a process**
“But even then clear routines are needed for how to do it. Then we may need to adapt them gradually when you notice that this does not work for the organisation. But when they have nothing at all when implementing for the entire habilitation services that’s a big shortcoming.” (No. 2, red.)
“Yes and a little bit how will it be here in our department how should we… uh you have to kind of break it down and see what is it that we are focusing on in this what should we concentrate on.” (No 7, yellow.)
“Eh I think it has felt like it has been… eh well thought-out and kind of step-by-step. Eh well thought-out so that it can so that it can kind of be of use for us eh who eh work in like the [computer] systems and with the programmes of care.” (No. 6, green.)
**C. Prerequisites to implementation**
“Eh and I think that’s kind of consistent with all the new processes here that uh you want people and you want to start things but you kind of underestimate the time”. (No. 1, red.)
“Eh and fo- for those who have had it a little easier to like switch to the new it has gone well but for those who think it’s a little more difficult it will be it still is really difficult because you remain in this thing you got from the beginning.” (No. 3, yellow.)
**D. Organizational and social work environment**
“Eh so for me it has probably become more of a eh nagging remorse because I know I should do it and some pressure also in that ah I should be able to manage and we did after all received this much information.” (No. 1, red.)
“And there are quite a few new things both- ah eh that have happened here recently […]. So I’m also thinking about like how much how many new things eh can eh our colleagues and we handle at the same time?” (No. 4, yellow.)
**E. Outcomes of the new method**
“Because I don’t think I can write about the motor ability* eh in a good way. Even if the patient says something about it.” (No. 4, red.)
“Eh and then eh in our department it has gotten a lot that you’ve sent a Messenger** then to others [asking] can you add to (laughter) the information collection template with your ongoing eh efforts and eh with information about what you know.” (No. 4, yellow.)
“But when we use it I think it’s good it facilitates and you have a better overview of the treatment efforts.” (No. 2, green.)
**F. Management and leadership**
“Eh because it becomes it feels like ah every department has been a bit left to themselves like do this. Or and that you’ve had to figure out like ah yourselves how to t- how to work in the department regarding implementing. And that feels I think not so good. And that it ah… Unclear.” (No. 5, red.)
“It’s always this when you have to sit with your calendar and prioritize because there is never anyone who will say like yes but the implementation work is more important than your scheduled patients, you should cancel on patients… So then you have to set the schedule in advance.” (No. 1, yellow.)
“Eh, well yes I feel that the support has been good eh in the sense that the planning has been distinct eh our manager has taken charge in that we should implement it as well.” (No. 6, green.)
**H. Implementation activities**
“But you would have needed in this too like ah but now now let’s take these few days and familiarize ourselves with this eh care plan and then you get another two days to review the patients you have and see what their care plans look like and work on them.” (No. 3, yellow.)
“Eh ah well both with these workshops and that we discussed it in both larger and smaller groups but also in like the workplace meetings and on an organizational day*** but it feels like it has been a procedure and a planning as well that has been clear I think.” (No. 6, green.)
**I. Lack of fidelity**
“Now it feels like there is a greater risk that if you don’t know exactly what to do with the information collection template then uh you do nothing at all.” (No. 1, red.)
“No, no so eh yes no but it’s again like I said eh so we’ve had these workshops bu- but not like hands-on now we work according to this but no.” (No. 7, yellow.)
**K. Differences in the implementation process**
“Ah, but also it feels in a way I feel sorry that uh it’s been so different at the departments”. (No. 5, red.)
“So now like what you said [No. 1] also about responsibility so both like who’s responsible for updating an- an- an- like in practice but also who’s responsible for the implementation can perhaps be a bit different in different departments. It sounds like in some cases it’s been the eh social workers who showed others eh in other cases it’s been maybe the manager who kind of announces what’s going on.” (No. 4, yellow.)

*Notes*: Each concept shows an exemplifying quote from each of its main colour categories (red, yellow, and green). Some concepts did not contain all categories, hence shows only two examples

*Reference to teamwork when respondent has difficulties describing the patient’s ability as it is not related to their own professional expertise

**Encrypted message sent through the digital medical records system

***Recurring joint sessions for competency development within the organization

Concepts A and B can appear similar in name. However, A represents when employees described that the execution of the implementation was inadequate. In comparison, B consists of employees’ reflections trying to make sense of the principles of the current implementation. For example, negative utterances can be found in both concepts, however those in B concern the process of organisational change like individual challenges to adapt to the new method or potential declines in patient care.

J and L were considered secondary findings not directly related to the implementation process. G was largely made up of statements concerning the method being implemented and not the implementation itself. These three concepts are therefore not discussed within the scope of this article.

### General disappointment and difficulties with momentum and appropriate supportive activities

Negative statements were the most common during the interviews while positive statements were rarer. The most common concept (A), with two thirds of the statements being negative, was experiences of the implementation process being insufficient. For instance, shortcomings were mentioned concerning time, support and the implementation activities that were provided. Employees felt there was a lack of initial preparation, of information and of established routines. The implementation was also considered insufficiently coordinated and the process too slow and protracted.

Opinions of the general process of implementation were however mixed (B). Respondents gave examples of events and aspects of the process that they had experienced as helpful. However, the difficulties of receiving effective support were also discussed. Positive expectations of the implementation and the new method at the start were shown to degrade over time as problems, disappointments and uncertainty arose. There was a contradiction between the employees’ need for direction and their wish to make local adaptations of the method to better suit the complexity of the seven different care settings.

### Differing contextual factors, implementation activities and fidelity

Concept C showed how different contextual conditions impacted the outcome of implementation. This concept lacked positive experiences, and instead was dominated by mostly negative and some neutral ones. One prominent contextual condition was the lack of time given to learning and trying out the new method. Employees felt that the time needed was routinely underestimated by management and that this always put implementation in competition with their ordinary clinical practice.

Another condition was the individual differences of the employees. Factors such as already having a well-established knowledge of previous routines or not being technically skilled were considered obstacles to adopting the new method. These groups of employees seemed to need significantly more supportive activities than their counterparts. Similarly, depending on the departments’ clinical focus and history there were differences in their uniquely established ways of working as well as varying amounts of administrative skills. This having the potential to make implementation either easier or harder.

The need for more support was also apparent regarding adoption of the method in some clinical areas due to employees’ scepticism to the fit of the method within their patient population. This was evident concerning patients with comorbid conditions, when complex teamwork is involved and with patients whose supportive network lacks initiative or is unfamiliar with how habilitation care operates.

The obstacles above reappeared in the concept concerning fidelity (I). Issues like the complexity of the method, uncertainty about correct usage and how time consuming it was to learn were discussed in both interviews. Together with experiences of technical difficulties with the software and lack of immediate support when needed these were all reasons for employees to carry on as before instead of practising the new method. Notably, this concept was made up almost entirely of negative statements. In contrast, the concept describing organized implementation activities (H) contained no negative statements at all. Activities that were mentioned as important to a successful implementation were written instructions complemented with personal support when needed, the use of test-users beforehand, and having a recurring and uninterrupted work time scheduled for instruction, practice, and joint discussions with colleagues.

### Work environment and leadership

Another concept (D) that was almost entirely made up of negative statements revealed the impact that the implementation process had on employees’ organisational and social work environment. Feelings of uncertainty, confusion, frustration, stress, exhaustion, and remorse over not doing one’s job well enough were commonly mentioned in both interviews. Experiences of lowered self-confidence in one’s professional capabilities were described as a result, and notably older employees were seen as being at greater risk for reduced self-confidence than younger ones. Feelings of ethical stress were also described in reference to the extra amount of responsibility that employees put on themselves to compensate for perceived failings in care. The implementation was described as contributing to an increased workload and one respondent (no. 4) questioned the number of changes that employees were able to handle at once (see Table 1).

The importance of a manager to actively lead the implementation process was strongly evident in the interviews. Statements ranged from mostly neutral to negative but were also accompanied by some positive experiences (F). A need was described for managers to exert active control over the process and lead in a clear direction to enable the implementation to move forward. This was done for instance by encouraging discussion in the team, forwarding questions to upper management, and clarifying employees’ respective responsibilities. However, uncertainty regarding roles and responsibilities were common. There seemed to be a lack of governing guidelines and the feeling of being left alone with the responsibility for implementing the new method was described by several respondents. Employees were disappointed in management’s failing to not only appoint enough time but also in keeping it sacrosanct and not up to the employee’s own conscience to defend.

### Differences in the implementation process

According to the respondents, the implementation process was experienced differently in the different departments. There was some understanding that the heterogeneity of the departments required a complex and differing strategy. For instance, in some departments, the new method affected routines of teamwork, but for other departments where teamwork was already an integral part of the work process not much change was needed. At the same time, there was an obvious interest to know how the others’ experiences of the implementation had been, and the reasons for the strategies differing between the teams.

### Experiences of outcomes from the new method

The larger collection of positive experiences was found in relation to the outcomes that the method had on work effectiveness and patient care (E). Later disappointments with the implementation notwithstanding, employees’ first impression of the method was positive. When in use it was perceived as more effective, more structured, practically helpful and giving a better outline of the patient’s care. Several respondents described the new method as more conducive to teamwork. However, this was not only a positive aspect since it also exposed when teamwork was not functioning properly from the beginning.

## Discussion

The aim of the present study was to investigate employees’ experiences of an ongoing large-scale implementation and what they perceived as important to succeed in a complex organisation within the public health care sector. Special interest was taken in finding patterns of perceived strengths or challenges that affected the implementation process.

Main findings show that although the implementation was not entirely a negative experience this was the most common type of statement while positive statements were rare. Employees were in general unsatisfied and felt the implementation was insufficient. This is in line with previous studies showing that many implementations end in failure [6] and that implementation in disability care is particularly complex and is experienced by managers as challenging [13].

In contrast to the negativity, nuances can be seen thanks to the novel method of colour coding. For instance, the concept concerning implementation activities (H) contained no negative statements at all and the concept concerning effects of the new method (E) was predominantly positive. Also, during the process of implementation there were expectations of the method and its implementation that initially were positive. Employees seem positive to the new method and to the implementation activities they took part in. Simultaneously, there is also evidence of a lack of fidelity (I). Due to the study’s research design, we cannot determine to what degree the implementation can be considered successful. One possible explanation is that although employees could see the new method in a positive light, due to insufficient implementation they rarely used it. In reference to the continuum described by Greenhalgh and colleagues (2004) one might say a dissemination but not a sufficient implementation have occurred [19]. In other words, due to planned strategies we see an active spread of the new method, but practices are not integrated within the organisation. Notably, most positive experiences were found in relation to work effectiveness and patient care. As both areas are closely related to the outcome of employees’ clinical work, our understanding of this can be aided by classic theories like those of intrinsic motivation and self-determination theory. Results could be seen as an example of how individuals are more motivated when an activity aligns with their intrinsic goals [20, 21]. This is echoed by previous research where managers noted that employees were positive to change when it was in the patients’ best interest [13]. Further similarities can be seen when comparing the present results with those of Granberg and colleagues (2021) [13]. Both managers and employees in the field of disability care struggle with balancing implementation with ordinary duties, experience a lack of sufficient time and wish for clearer direction and priorities from managers. Parallel results have also been found when investigating employee experiences in psychiatric care [22]. These supportive functions can be seen as the content of the package of implementation policies and practices described by Klein and Knight (2005) and is one of the key factors necessary for successful implementation [6]. Although they were experienced as missing in the implementation presently studied, they are evident in other guidelines concerning implementation. For instance, the CFIR (Consolidated Framework for Implementation Research) mentions available resources as a factor of the inner setting domain that influence implementation. It also mentions important aspects of leadership in the roles sub-domain [23]. The ERIC taxonomy (Expert Recommendations for Implementing Change) mentions strategies like providing technical assistance and expert consultations [24]. Finally, the QIF (Quality Implementation Framework) describes the on-going responsibilities of the support system to ensure quality implementation [25]. The need for support returned in concepts regarding both implementation activities (H), prerequisites to implementation (C) and lack of fidelity (I), suggesting a relationship between them. This is similar to the Value Equation described by Von Thiele Schwartz and colleagues (2019) where implementation strategies that optimize the organisational context can be seen as means to increase fidelity [9]. A contribution that the present study brings to this equation is how the employees subjected to implementation processes experience these factors. Differing contextual factors were experienced as either helping or hindering implementation (C), such as individual technical skills or a departments’ clinical focus or history, depending on the needs of selected groups. In a complex and heterogeneous organisation this brings a need for different implementation activities that are made available either by choice or that can be adapted to be larger or smaller as needed. This is also in line with the Value Equation and with previous research that has shown how tailoring interventions improve performance [26–28]. However, this means that in large complex organisations employees will experience the implementation differently depending on their different perspectives. In the present study when no logic was seen to explain this, nor any information given, these differences gave rise to scepticism. Instead, leaders need to be transparent in their strategies and communication.

One of the larger concepts that was made up almost entirely of negative statements (D) shows another main result which is the amount of strain that the implementation process put on employees’ organizational and social work environment. Given the many changes in health care at present [2, 4, 5] employees become subjected to many different implementation processes. Given the simultaneous shortage of health care staff [11], together with prevalent stress [12], it is relevant for decision makers of future implementations to take these results into account. Given that many implementations end in failure [6] it is reasonable to suggest that poorly executed implementations could be a contributing factor to employees leaving the health care sector. In an already strained situation, the question begs to be asked whether all implementations really are necessary. The Public Health Agency of Sweden, who base their guidelines largely on the Quality Implementation Framework, notes that the first ten out of fourteen steps entail preparations as well as identifying and handling factors, which may obstruct or promote implementation [29]. This puts a lot of responsibility on leaders and many studies also show the importance of leadership [6, 25, 30]. As Klein (2005) puts it, managerial patience is one important factor [6]. But also, without strong, convincing, informed, and demonstrable support “employees are likely to conclude that the innovation is a passing managerial fancy: Ignore it and it will go away”. Yet as stated by Ovretveit (2010) associations in the different studies are weak [31]. What exactly a leader should do is less certain, and results may not be generalisable to other contexts. The current study’s results on leadership were also mixed (Concept F) with experiences of both negative and supportive nature. A common topic though was the feeling of being left alone with the responsibility for the implementation and the ethical dilemma of having to prioritize between taking time to learn the new method and caring for patients. Regarding the psychosocial effects noted above, one important action for leaders seems to be the ability to protect the employees from unnecessary strain. To take responsibility, lead in a clear direction and make clear priorities concerning employees’ time was also needed by managers leading implementation processes from *their* managers [13]. This again shows the complex interaction between implementation and context as many of the supporting conditions on local levels are the results of leaders’ actions at higher levels [31]. More examples of facilitating leadership behaviors from the present results are encouraging team discussions, forwarding questions, and clarifying employees’ respective responsibilities.

The present study has more implications for practice that leaders should consider. Having test-users beforehand can be a way to protect employees by limiting their exposure during the first stages of the implementation. Information and routines need to be established early on. As the organisation learns these can be adapted, but coordination is needed so that the process does not become too protracted. Written instructions are one type of support shown in the results as being appreciated by most respondents. However, instructions must be accompanied by extra support that are readily available to certain groups or individuals when context requires it. Sufficient time to accommodate both instructions, trying out the new method as well as team discussions are further examples of appreciated supportive functions. But if this is put in competition with ordinary clinical practice it can be experienced as stressful.

### Limitations and methodological perspectives

The study has some limitations. Due to a small and self-selected sample of respondents, it is uncertain whether the results can be generalized to the remaining employees or to that of other organisations. It is possible that experiences represented are limited to those who strongly feel they have something to say about the implementation, thus being either very positive or very negative compared to their co-workers. However, representation was established from each of the departments affected by the implementation and from a wide spectrum of the clinical professions in question, which likely is the more important aspect for achieving a comprehensive depiction of how different employees within the same organisation are affected by the implementation. To achieve this focus groups needed to be held on two separate occasions, which led to the latter group being substantially smaller than the first (*n* = 2 vs. *n* = 7). The optimal number of participants in focus groups are usually around six, but it is also possible to have as few as three [16]. Having only two participants could have led to differing responses in comparison to the first group. However, the discussion between the two participants was an important contribution as it enabled data collection from each of the seven departments. Efforts were made to use the same procedure and questions in both groups. In neither group were the participants familiar with each other or with the moderator. The length of the second focus-group interview was shorter, but this was expected since there were fewer participants to respond to the same questions.

Even so the results represent the experiences of only one complex organisation. Similarities of the results to those in previous studies [13, 22] gives reason to suggest that they are representative of organisations in public health care, but perhaps only to disability and psychiatric care. Contextual factors, such as the organisation’s clinical focus or history, need to always be considered and decisions on implementation made accordingly.

The internal validity of the results is strengthened by the fact that the authors come from different occupational professions and backgrounds and were both involved in creating consensus in all stages of analysis. The first author (MS) had current clinical knowledge from within the organisation while the second researcher (UL) was an outside researcher connected to the university.

The novel approach to content analysis also brought another layer of information to the data, thus getting extra output from a small sample. The mixed-method element gave an explorative depth in content while at the same time giving a systematic overview of the patterns of data. The quantitative calculations showed both the size of the concepts which corresponded to how common the different topics were, as well as the distribution of positive and negative statements which showed where employees experienced the strengths and weaknesses within each concept. In addition, early detection of statements not related to the research questions gave the opportunity to belay focus on certain concepts. These can instead be analysed at a later stage. Future research can benefit from this novel approach when analysing similarly complex and diverse materials of qualitative data.

## Conclusions

Implementation processes within large and complex health care organisations are experienced as challenging for employees. Key facilitators are available support functions, clear leadership and time that is sufficient and kept sacrosanct. Consideration needs to be given to the fact that helping or hindering contextual factors influence each other. At the same time leaders need to communicate how and why implementation processes may be experienced differently in a large and complex health care organisation. The impact that organisational change has on the work environment should be acknowledged from the initial stages of planning and throughout the implementation process. The field of implementation research would benefit from more research on how failed implementations negatively impact employees’ social and organisational work environment to see if significant correlations can be discovered.

### Electronic supplementary material

Below is the link to the electronic supplementary material.

**Supplementary Material 1:** Details on the implementation

**Supplementary Material 2:** Standards for reporting qualitative research (SRQR)*

**Supplementary Material 3:** Interview guide

**Supplementary Material 4: Table 1.** Concepts according to size with distribution of colour categories

## Data Availability

The dataset presented in this article are not readily available because the study used qualitative data generated from interviews and respondents’ integrity is paramount. Anonymous samples of the datasets used and analyzed during the study are available from the corresponding author on reasonable request.

## Abbreviations

GDPR: General data protection regulation
SRQR: Standards for reporting qualitative research
