# Supplementary material for: Employees’ experiences of a large-scale implementation in a public care setting: a novel mixed-method approach to content analysis
Source: BMC Health Serv Res. 2024 Jan 18;24:107. doi: 10.1186/s12913-024-10560-9 (PMC10797789; doi:10.1186/s12913-024-10560-9)
Supplement: Supplementary file 1 — Supplementary Material 1: Details on the implementation [file 12913_2024_10560_MOESM1_ESM.docx]

**Additional file 1**

**Details on the implementation**

The change that is being implemented by the organization is a new method of documenting and revising patients’ care plans. The previous method was technically limited so that the old documentation could not be updated but had to be completely replaced when changes occurred. This made it reliant upon yearly renewals during large follow-up meetings with patients. The meetings were often time consuming or took place at a much later date than needed. This sometimes led to patient records not being sufficiently updated or to uncertainty as to what planning had been agreed upon. The new method includes a new digital template for documenting patient information and decisions about care. This template gives employees the ability to make continuous updates as patients’ care and context changes. The goal is a clear and up-to-date summary of the multifaceted needs of patients and the collection of corresponding health care measures that have been decided upon.

The implementation first started with a select occupational group (social workers) in the beginning of 2021. Introduction of the new template and associated checklists were made during a seminar. This pilot-trial led to slight alterations of the template. The method was then considered viable for introduction to the rest of the organization in the fall of the same year. Introductory seminars, workshops to try the method in a trial-run program, as well as a joint organizational half-day for all employees, were held. Responsibility for further implementation was placed on first-line managers. Repetitions of the workshops, distributions of locally adapted routines or checklists, team discussions, or other implementation activities like the presence of locally appointed ambassadors who were available for individual support, were therefore made available in some departments but not others. At the time of data collection in the spring of 2022 there was no clear timeframe for when the implementation was expected to be completed, nor was there a clearly defined plan for evaluation. However, this has later been attended to by management in a new phase of the implementation by reviewing what percentage of patients care plans have been updated using the new method.
