# Supplementary material for: Employees’ experiences of a large-scale implementation in a public care setting: a novel mixed-method approach to content analysis
Source: BMC Health Serv Res. 2024 Jan 18;24:107. doi: 10.1186/s12913-024-10560-9 (PMC10797789; doi:10.1186/s12913-024-10560-9)
Supplement: Supplementary file 3 — Supplementary Material 3: Interview guide [file 12913_2024_10560_MOESM3_ESM.docx]

**Additional file 3**

**Interview guide.**

Instructions to participants on how the interview will be conducted:

*I will be asking some questions for you to discuss about the implementation of the new working method to make patient care plans into a living document. The recording will be deleted immediately after the answers are transcribed.*

*Before we begin the interview, I want to inform you that your participation is voluntary. You can end your participation at any time during the conversation without giving a reason. The conversation will be recorded, and the recording will be stored securely so that only researchers in the study have access to the data.*

- How do you feel that the implementation process for the new way of working with care plans has been so far?
- What is it like to work according to the new working method?
- How does the new way of working differ from how it was before?
- How has the support from the organisation been to start using the new working method?
- How has your working situation been affected by the new working method? For example, regarding workload or teamwork.
