# Supplementary material for: Employees’ experiences of a large-scale implementation in a public care setting: a novel mixed-method approach to content analysis
Source: BMC Health Serv Res. 2024 Jan 18;24:107. doi: 10.1186/s12913-024-10560-9 (PMC10797789; doi:10.1186/s12913-024-10560-9)
Supplement: Supplementary file 4 — Supplementary Material 4: Table 1. Concepts according to size with distribution of colour categories [file 12913_2024_10560_MOESM4_ESM.docx]

**Additional file 4**

| **Table 1: Concepts according to size with distribution of colour categories.** | | | | | | |
| --- | --- | --- | --- | --- | --- | --- |
| **Concept** | **Total**  **% (n)** | **Red**  **% (n)** | **Yellow**  **% (n)** | **Green**  **% (n)** | **White**  **% (n)** | **Blue**  **% (n)** |
| A. Insufficient implementation | 25 (88) | 67 (59) | 33 (29) | 0 (0) | 0 (0) | 0 (0) |
| B. Implementation as a process | 16 (54) | 15 (8) | 63 (34) | 17 (9) | 6 (3) | 0 (0) |
| C. Prerequisites to implementation | 9 (31) | 52 (16) | 35 (11) | 0 (0) | 10 (3) | 3 (1) |
| D. Organizational and social work environment | 9 (31) | 90 (28) | 3 (1) | 0 (0) | 0 (0) | 6 (2) |
| E. Outcomes of the new method | 9 (30) | 10 (3) | 3 (1) | 70 (21) | 10 (3) | 7 (2) |
| F. Management and leadership | 7 (26) | 35 (9) | 42 (11) | 19 (5) | 0 (0) | 4 (1) |
| G. Living document compared to traditional care plan | 6 (22) | 18 (4) | 18 (4) | 14 (3) | 45 (10) | 5 (1) |
| H. Implementation activities | 5 (19) | 0 (0) | 74 (14) | 26 (5) | 0 (0) | 0 (0) |
| I. Lack of fidelity | 4 (13) | 85 (11) | 8 (1) | 0 (0) | 8 (1) | 0 (0) |
| J. Consequential effect of implementation | 4 (14) | 21 (3) | 21 (3) | 14 (2) | 7 (1) | 36 (5) |
| K. Differences in the implementation process | 3 (11) | 9 (1) | 45 (5) | 0 (0) | 36 (4) | 9 (1) |
| L. Care delivery process | 3 (9) | 11 (1) | 0 (0) | 0 (0) | 22 (2) | 67 (6) |
| **All Concepts** | **100 (348)** | **41**  **(143)** | **33 (114)** | **13**  **(45)** | **8**  **(27)** | **5**  **(19)** |
| *Notes:* Red: Negative statement. Yellow: Neutral statement. Green: Positive statement. White: Unspecified. Blue: Statement related to insights that occurred because of the implementation process. | | | | | | |
